# Dose Tapering and Discontinuation of Biologic DMARDs in Axial Spondyloarthritis: A Narrative Review (2023 SPARTAN Annual Meeting Proceedings)

**DOI:** 10.1007/s11926-024-01137-w

**Published:** 2024-02-09

**Authors:** Haseeb Chaudhary, Mohamad Bittar, Ansaam Daoud, Marina Magrey

**Affiliations:** 1 Department of Rheumatology, Case Western Reserve University, University Hospitals, Cleveland, OH USA; 2 Division of Arthritis and Rheumatic Diseases, Oregon Health and Science University, Portland, OR USA

**Keywords:** Axial spondyloarthritis, Biologic disease modifying anti-rheumatic drugs, bDMARDs, Tapering strategies, Tumor necrosis factor inhibitors, TNFi

## Abstract

**Purpose of Review:**

Limited data is available for tapering or discontinuation of biologic therapy in patients with axSpA who are in disease remission. The current review concentrates on published studies regarding dose tapering or withdrawal of biologics in axSpA.

**Recent Findings:**

Recent evidence in light of randomized controlled trials suggests that tapering of b-DMARDs is a feasible strategy to maintain remission or low disease activity in axSpA patients. TNF inhibitors were the studied biologics in most of these trials. The disease flare rates were comparable to those maintained on standard dose in most of these studies, although with variable tapering strategies and follow-up. Additionally, the duration of disease in remission prior to tapering, studied primary outcome, and flare definitions were heterogeneous. Female sex, HLA-B*27 negativity, high physician global score, and high CRP were negative predictors of successful tapering, but not consistently reported in all the trials. Although designed to address efficacy, there were no safety concerns with b-DMARD tapering. Withdrawal or complete discontinuation of biologics met with increased risk of flares compared to standard dosing.

**Summary:**

Tapering of TNF inhibitors may be feasible in certain axSpA patients with an acceptable disease state; however, discontinuation is not currently recommended owing to increased risk of flare. Future studies with axSpA patients with longer remission duration prior to taper and different doses and types of b-DMARDs may provide more guidance.

## Introduction

Axial spondyloarthritis (axSpA) treatment has considerably evolved in the past two decades. The availability of biologic disease-modifying agents (b-DMARDs) and newer synthetic disease-modifying agents (s-DMARDs) has fundamentally changed the treatment paradigm of axSpA [1]. Several b-DMARDs, including tumor necrosis factor-inhibitor (TNFi) and interleukin-17A inhibitor (IL-17Ai), have become standard of care for the treatment of axSpA along with Janus kinase inhibitors (JAKi) approved in recent years [2]. In addition to improving symptoms and reducing spinal inflammation [3, 4], studies have also reported significant improvement in patient-reported outcomes such as Short Form Health Survey (SF-36), EuroQol 5 Dimension 5 (EQ-5D), and Ankylosing Spondylitis Quality of Life scale (ASQoL) in radiographic axSpA (r-axSpA) in patients treated with TNFi (infliximab, etanercept, adalimumab, golimumab, and certolizumab) and IL-17Ai (secukinumab and ixekizumab). Moreover, work productivity and activity impairment also improved with biologic therapy in axSpA patients [5].

The benefits of b-DMARDs are offset by the concern of adverse events. A commonly posed inquiry from patients with axSpA experiencing sustained positive outcomes with treatment is whether discontinuation of the medication is feasible, considering the safety implications associated with the prolonged use of biologics.

Most axSpA patients are diagnosed in young adulthood, and economic evaluation of health interventions is important. According to US administrative data, patients with axSpA had approximately 4-fold higher mean total all-cause healthcare costs than the matched control population without axSpA, primarily driven by medication costs [6]. Conversely, similar studies have also shown higher healthcare resource utilization and medical costs in axSpA patients who prematurely discontinued therapy than those who were maintained on index b-DMARD therapy [7]. Although the 2019 ACR/SAA/SPARTAN guidelines for the treatment of axSpA conditionally recommended against discontinuation of biologics as the standard approach in patients with stable axSpA [8], tapering of biologics can be considered in prolonged stable disease with shared decision-making between the patient and the provider. It is important to note that the evidence for discontinuation and tapering strategies was limited and of low quality at time of formulating those recommendations. However, in 2022, ASAS-EULAR updated the recommendations for the management of axSpA and added a new recommendation to consider tapering bDMARDS in patients with sustained remission for a minimum of 6 months [9]. Treatment aims to induce clinical remission or low disease activity (LDA) utilizing various therapeutic regimens. The decision to start dose tapering after achieving remission is fraught with challenges. There is minimal guidance on how to de-escalate therapy for axSpA patients in disease remission compared to initiation and escalation of therapy for control of disease activity. There are several patient and disease factors to consider before opting for de-escalation of therapy, such as providers’ and patients’ perception of disease activity, prior response or failures to b-DMARDs, type of b-DMARD in use, and acceptance of low disease activity as opposed to complete remission with a balance of patient-reported outcomes. The current review focuses on whether it is possible and safe to taper or discontinue b-DMARDs in axSpA patients with LDA or clinical remission.

## Search Strategy

We reviewed all published data about dose tapering or withdrawal in axSpA studies. Our literature search was limited to articles published in English only. The search included key words such as “ankylosing spondylitis,” “axial spondyloarthritis,” “tapering,” “discontinuation,” “drug withdrawal,” “TNF inhibitor,” “IL-17 Inhibitor,” “Janus kinase inhibitors,” “biologics,” and “b-DMARD.” Additionally, 2019 ACR/SAA/SPARTAN and 2022 ASAS-EULAR management recommendations for axSpA were reviewed [8, 9]. Lastly, the references of these recommendations on tapering and discontinuation in axSpA were also screened for additional articles of interest. Articles with full text only were included for this review.

## The Current Evidence on Dose Tapering of Biologic DMARDs in Axial Spondyloarthritis

Initial heterogeneous studies reported several b-DMARD tapering strategies in terms of their efficacy for maintaining disease remission or LDA in patients with axSpA [10–12]. In a systematic review, eight studies [13], including one small pilot study and a randomized trial [14, 15], were analyzed. The percentage of patients maintaining LDA or remission after reduction of the TNFi dose was reported in five of the eight studies with variable follow-up (67–100%) [10, 12, 14, 16, 17]. However, the quality of evidence was very low because of the studies’ small sample size and observational nature. Since then, several randomized controlled trials on tapering strategies of TNFi in axSpA have been reported [18–26]. These trials differ in tapering strategies, including duration of disease remission before drug tapering, dosing intervals, measured disease activity indices, and follow-up duration (Table 1). Most of these trials were conducted in Europe and two in China [18, 22].

**Table 1:** Randomized clinical trials reporting different strategies and outcomes utilizing various TNFi b-DMARDs

Study	Design	Location	*N*	Induction	Strategy	Outcome	Follow-up	Results
Cantini et al., 2013 [14]	Randomized, parallel-group, single center	Italy	42, axSpA	25 ± 11 months	ETN q2Wk vs. standard dose	BASDAI < 4, or no EMM	22 months	Remission in 86.3% vs. 90.4% in standard dose group
ANSWERS Yates et al., 2015 [21]	Randomized, controlled, non-inferiority	UK	47	26 weeks	ETN 25 mg q Wk vs. standard dose	Loss of remission defined by increase in BASDAI >.2 or 50% increase in baseline + pain VAS > 2	24 weeks	Remission in 52% vs. 83.3%
Li et al., 2016 [18]	Randomized, single center, open label	China	43, axSpA	4 weeks	ENB 50 mg q4Wk followed by 25 mg q 4Wk vs. standard dose	Mean BASDAI	8 weeks	Mean BASDAI 1.42 vs. 1.40 in standard dose group
C-OPTOMISE Landewe et al., 2019 [19]	Randomized, controlled, parallel-group multicenter	Europe	303, axSpA	48 weeks	CZM 200 mg q2weekly vs. CZM 200 mg q monthly	ASDAS < 1.3	48 weeks	Flare-free survival 79.0% vs. 83.7% in standard dose group
REDES-TNF Gratacos et al., 2019 [20]	Randomized open-label clinical trial	Spain	113	6 months	TNFi dose spacing by 50% vs. TNFi standard dose	BASDAI < 4,PhGA < 4 + PGA < 4 and nocturnal axial pain VAS < 4	52 weeks	81.3% vs. 83.8% in standard dose group (non-inferior)
Zhang et al., 2020 [22]	Randomized, multicenter, open label	China	235	12 weeks	Group A (remission i.e., ASDAS < 1.3) Group B (LDA ASDAS > 1.3–< 2.1) ENB Tapered to 25 mg q4 weeks vs. standard dose	ASDAS < 1.3 group A ASDAS (< 2.1 > 1.3) group B	48 weeks	Flare-free survival 91.0% and 83.3% in both groups with stepwise tapering
DRESS_PSMichielsens et al., 2022 [23••]	Randomized, controlled single center, open label	Netherlands	122, (axSpA 58)	> 6 months	Q3monthly Stepwise taper of TNFi with spacing of dosing intervals except for IFX with reduction in dose	Mean ASDAS, PASDAS	12 months	Mean ASDAS 1.34 (0.87) vs. 1.21 (0.61) in standard dose Mean PASDAS 1.60 (1.26) vs. 1.63 (0.98) in standard dose
Ruward et al., 2022 [24]	Randomized, open label	Netherlands	160	6 months	Phase 1—ETN dose reduction by 50% vs. standard dose Phase II—ENT discontinuation if maintained MDA vs. standard dose	ASDAS < 2.1	18 months	55% maintained MDA vs. 65% in standard group
BIODOPT Uhrenholt ed al., 2022 [25]	Randomized, open-label	Denmark	95 (axSpA 36)	> 12 months	TNFi spacing with reduction of dose by 50% vs. standard practice	50% dose reduction and ASDAS at 18 months	18 months	Mean ASDAS 1.46 (0.12) vs. 1.3 (0.11)
GO-BACK Weinstein et al., 2023 [26]	Randomized, open-label	International	188	10 months	GLM q2mo vs. standard dose compared to PLB	Flare free survival i.e., ASDAS < 2.1, BASDAI < 2 points	12 months	68.3% taper group vs. 84.1% in standard ( not significant)

*EMM* extra musculoskeletal manifestation, *VAS* visual analogue scale, *PhGA* physician global assessment, *PGA* patient global assessment, *TNFi* tumor necrosis factor inhibitor, *m* month, *w* week, *BASDAI* Bath Ankysloing Spondylitis Disease Activity Index, *ASDAS* Ankylosing Spondylitis Disease Activity Score, *PASDA* psoriatic arthritis disease activity score, *LDA* low disease activity, *VAS* visual analogue scale, *ENB* Enbrel, *ETN* etanercept, *CZM* certolizumab, *GLM* golimumab

An earlier randomized trial (RCT) [14] included patients who had achieved remission using etanercept and randomized them to standard dose treatment with spacing of the dose interval in the tapering group. It showed that nearly 90% of patients in the latter group maintained remission with a long-term follow-up of 22 months. The following trial by Yates et al. [21] failed to show the non-inferiority of dose reduction compared to standard dosing. Nearly 50% of the patients in the taper group maintained clinical response based on BASDAI score compared to 80% in the standard group. However, it is worth mentioning that patients included in this study were not in disease remission for a specific duration before randomization, which may have accounted for the negative result. The majority of the subsequent RCTs outlined in Table 1 suggest that tapering of b-DMARDs is a feasible strategy and non-inferior to standard dose in terms of maintaining remission in both axSpA and non-radiographic axial spondyloarthritis (nr-axSpA), the latter evidenced by fewer studies. All of these trials investigated the dose reduction of the TNFi class. There is no data about the dose reduction of other b-DMARDs used for axSpA in randomized trials.

## Tapering Strategies with Reported Risk of Flare

Several tapering strategies have been investigated in observational and subsequent randomized trials (Table 1). These strategies include increasing the dosing interval, referred to as spacing, or reducing the administered dose simultaneously or subsequently. The tapering regimens were either based on a fixed dose, one-step approach as observed in earlier trials [14, 18–21] or carried out in a step-wise fashion in recent studies [22, 23, 25]. In the DRESS-PS trial, patients experienced dose interval spacing of a TNFi based on a pre-defined protocol, followed by further tapering if they maintained LDA. The tapering consisted of 3-monthly steps (66%, 50%, 0%), with re-intensification in the event of a flare. At 12 months, 72% of patients in the tapering group were successfully tapered, of whom 28% discontinued their TNFi. The cumulative incidence of flare was 85% in the tapering and 78% in the no-tapering group (*p* = 0.32), concluding that stepwise TNFi dose reduction strategies seem feasible in axSpA [23••]. One trial by Zhang et al. [22] divided patients with clinical remission and LDA into step-wise tapering or delayed tapering arms (among patients with LDA) with dose reduction every 12 weeks until discontinuation. Although LDA is an acceptable alternative to complete remission in some patient populations with axSpA, the flare rates were slightly higher in this group than those in clinical remission prior to dose tapering. Moreover, the delayed tapering strategy in patients with LDA did not differ in flare-free survival compared to step-wise tapering. In comparison, in a treat-to-target (T2T) step-wise tapering in a randomized trial of 122 patients [23••], the proportion of patients with LDA among the taper group was non-inferior to standard dosing of TNFi, with a 53% reduction in daily-defined dosage in the former group with a follow-up of 12 months. Another prospective study from Denmark [27••] evaluated a disease activity-guided stepwise tapering-to-discontinuation scheme at 16-week intervals in patients with LDA. This study showed that half of the patients who experienced flare while tapering were on one-third or less of the standard dose. Nearly half of the patients could successfully taper TNFi and maintain remission after 2 years of follow-up, whereas discontinuation was not feasible. In a nationwide cohort of 776 axSpA patients from the Korean College of Rheumatology Biologics Registry (KOBIO), heavy tapering (< 50% of full dose) was associated with reduced odds of maintaining inactive disease compared to standard dosing, while mild tapering (50–99% of full dose) was not [28].

Overall, the step-wise taper strategy does offer an individualized approach with the advantage of reinstituting the previous dose in case of a relapse. There remains a paucity of data comparing different tapering strategies to achieve flare-free survival. Future studies directly comparing different strategies may provide more evidence of tapering efficacy and safety. There is also a need for consensus regarding optimal tapering strategies and time spent in remission with a standard dose before the de-escalation of therapy. In a Chinese study by Lee et al., [18] axSpA patients were included 4 weeks after induction with TNFi and showed no difference in mean BASDAI in the taper arm compared to standard dosing. However, most other trials included patients after at least 6 months of maintenance dose. The recent update by EULAR recommendations also makes a conditional recommendation to consider tapering of b-DMARD therapy in patients who have been in remission for 6 months, as defined by ASDAS < 1.2 [9].

## Effect on Function and Quality of Life Patient-Reported Outcomes

Patients’ perception of disease activity or concern for adverse outcomes before initiation of tapering is a critical aspect of its success. Studies have shown that discordance between physician and patient global assessments can make shared decision-making challenging [29]. In addition, the prospect of less frequent injections affecting patients’ quality of life is not perceived as impactful by axSpA patients [30]. A few studies have looked into the functional and quality of life measures in the conducted trials of b-DMARD tapering. Most other RCTs reported similar functional and QoL patient-reported outcomes between the studied groups. In a study by Michielensas et al., the mean function and quality of life as reported by the Health Assessment Questionnaire (HAQ), Bath Ankylosing Spondylitis Functional Index (BASFI), and Ankylosing Spondylitis Disease Activity Score-Health Index (ASAS-HI) did not differ significantly between the group on standard therapy vs. taper [23••]. In the previously mentioned Danish study [27••], patients in tapering arms had statistically significantly higher HAQ and Bath Ankylosing Spondylitis Metrology Index (BASMI) at 2-year follow-up. However, all these were of numerically minor magnitude. Previous studies reported no statistically significant differences in parameters such as C-reactive protein (CRP), HAQ, BASFI, and BASMI [14] when comparing patients on full dose vs. patients on reduced dose. At 48-month follow-up in the C-OPTIMISE trial [19], patients on standard and tapered doses were statistically significantly different from the placebo group in disease activity and functional index such as BASFI.

## Safety of Tapering

Another concern among the patients is the high risk of serious infections with the continuation of b-DMARD therapy. A previous meta-analysis addressed this specific concern. Studies were included if rheumatoid arthritis or spondyloarthritis patients were in remission or in a LDA state [31]. This meta-analysis evaluated 13 controlled trials: 9 of rheumatoid arthritis and 4 of spondyloarthritis. Serious infections occurred in 1.7/100 patient-years for those with “tapered” therapy compared to 2.6/100 patient-years in those who maintained a steady dose and no attempts at tapering. There was no difference in the risk of serious side effects or malignancies or cardiovascular events or deaths in RA and spondyloarthritis patients with tapering. Although plausible, the seeming advantage of dose tapering for fewer adverse events has not been evident in these trials. Most trials were designed to assess efficacy and underpowered to address safety with limited follow-up.

## Factors Associated with Successful Tapering of b-DMARDS in axSpA

Selecting appropriate patients for successful tapering is as vital as selecting a suitable strategy. The studies mentioned above have also investigated several patient and disease factors to predict the success of b-DMARD tapering. Unfortunately, the association of reported clinical indicators with successful tapering is heterogeneous. Various studies have reported female sex, human leucocyte antigen (HLA-B*27) negative status, high physician global visual assessment scale (VAS), CRP, ASDAS, Canadian Spondyloarthritis Research Consortium (SPARCC) erosion scores, and severe sacroiliitis to be negatively associated with successful tapering. In a Danish study by Wetterslev et al. [27••], 109 axSpA patients in remission for at least 1 year on standard or lower doses of TNFi, lower physician global VAS was the only independent predictor of successful tapering in patients who were on standard TNFi doses prior to taper (OR = 0.79;95% CI 0.64–0.93). In the analysis of the entire cohort on variable doses of TNFi prior to taper, low physician global VAS, low SPARCC sacroiliac structural joint damage (SSS) score, and current smoking were the reported independent positive predictors of successful tapering. All the patients that flared after tapering had significantly higher HAQ, BASMI, CRP, and more tender joint counts at baseline. In the ABILITY-3 trial [32], post hoc analysis showed lower ASDAS predicted maintenance of remission. Another post hoc analysis of the C-OPTIMISE study [19] found that HLA-B*27 negativity was associated with a higher chance of disease flares in the treatment group. However, it is important to note that the number of flares in the treatment group was small. In the REDES-TNF trial [20], BASFI and the type of TNFi were linked to successful tapering and LDA after 1 year of treatment. There was no significant difference among different dose groups. Additionally, the patients who did not reach LDA at 1 year had higher CRP levels. However, according to that study, using the CRP alone to predict outcomes was inaccurate, given its poor predictive value. In another study by Zhang et al. [22], high levels of CRP, severe sacroiliitis, and discontinuation of TNFi predicted disease flares in axSpA patients who were in clinical remission before TNFi taper. No individual factor associated with an unsuccessful taper was reported in the parallel group with LDA. The overall flare rate was higher in the LDA group compared to the cohort in clinical remission with a similar tapering strategy in this study.

In summary, the potential predictors of success/failure of the tapering strategies have yet to be consistently identified in the available data. The approach to discontinuing or tapering may depend on the patient and the clinical context.

### Negative Predictors of Tapering

1. Female sex
2. HLA-B*27 negativity
3. Higher physician global score
4. High CRP level

## Current Data on Discontinuation of b-DMARD

One of the main uncertainties in axSpA is the duration of treatment and whether the possibility of treatment discontinuation exists. Most of the initial data were derived from post hoc analyses and observational studies, and it was not until 2018 that international randomized RCTs studying b-DMARD discontinuation started to emerge.

Studies to date have looked mainly at TNFi (4 etanercept, 2 adalimumab, 2 infliximab, 1 golimumab, 1 certolizumab, 1 any TNFi), with only one study discussing IL-17i (ixekizumab). No discontinuation studies have been reported on JAKi.

Initial studies were mainly open-label observational studies, where patients were followed for some time (36 to 52 weeks) after they had completed a previous phase of biologic treatment as a part of a clinical trial. TNFi were discontinued instantly without being tapered. The definition of a flare was based on BASDAI (BASDAI ≥ 4 or increase of 2 units compared to baseline) in all of the studies, except for one [33], which defined a flare based on loss of the ASAS40 response that was previously achieved. Similar to the studies mentioned above for tapering, each study had a different definition (BASDAI vsASAS responses) of what constitutes remission or low disease activity based on which the TNF inhibitor was discontinued (Table 2). The sample size was small (range of 17–42 patients), and most patients met the classification of radiographic axSpA or ankylosing spondylitis (AS). One study included nr axSpA [33]. The flare-up rates were very high, ranging from 76 to 100%, and were consistent among AS and nr-axSpA patients. One RCT in China in 2012 [39] studied Etanercept withdrawal in 111 patients with AS (r-axSpA). This trial followed patients for 52 weeks, and 79% had a flare based on BASDAI definitions (> 2 unit increase in BASDAI or relapse to 80% of BASDAI before treatment).

**Table 2:** Studies reporting biologic withdrawal in axial spondyloarthritis

Study	Biologic	Design	Location	N	Timing	Strategy	Outcome*	F/up	Results
Brandt et al., 2003 [34]	Etanercept	Obs	Germany	26	3 m	Instant	Flare (BASDAI)	36 w	100%
Baraliakos et al., 2005 [35]	Infliximab	Obs	Germany	42	36 m	Instant	Flare (BASDAI)	52 w	98%
Deng et al., 2012 [32]	Etanercept	RCT	China	111	2.5 m	Instant	Flare (BASDAI)	52 w	79%
ESTHER, 2012 [36]	Etanercept	Obs	Germany	17	11 m	Instant	Flare (BASDAI)	52 w	76%
Haibel et al., 2013 [31]	Adalimumab	Obs	Germany	24	12 m	Instant	Flare (ASAS40)	52 w	79%
ABILITY-3, 2018 [32]	Adalimumab	RCT	International	153	28 w	Instant	Flare (ASDAS)	40 w	52.9%
REMINEA, 2019 [37]	Infliximab	Obs	Spain	36	6 m	Instant	Flare (BASDAI)	48 w	58%
C-OPTIMISE, 2020 [19]	Certolizumab	RCT	International	104	48 w	Instant	Flare (ASDAS)	48 w	79.8%
COAST-Y, 2021 [24]	Ixekizumab	RCT	International	53	24 w	Instant	Flare (ASDAS)	40 w	45.3%
Wetterslev et al., 2022 [27••]	Any TNFi	Obs	Denmark	109	12 m	Taper	Flare (BASDAI)	96 w	99%
GO-BACK, 2023 [27••]	Golimumab	RCT	International	63	10 m	Instant	Flare (ASDAS)	52 w	66.1%
RE-EMBARK, 2023 [38••]	Etanercept	RCT	International	119	24 w	Instant	Flare (ASDAS)	40 w	74.8%

*F/up* follow-up period, *Obs* observational, *RCT* randomized controlled trial, *TNFi* tumor necrosis factor inhibitor, *m* month, *w* week, *BASDAI* Bath Ankysloing Spondylitis Disease Activity Index, *ASDAS* Ankylosing Spondylitis Disease Activity Score

*Flare definitions based on studies: Brandt et al. and Baraliakos et al. (BASDAI ≥ 4 and physician’s global assessment ≥ 4); Deng et al. (> 2 unit increase in BASDAI or relapse to 80% of BASDAI prior to treatment); ESTHER (> 2 unit increase in BASDAI); Haibel et al. (loss of ASAS40 response); ABILITY-3 (ASDAS ≥ 2.1 at 2 consecutive visits); REMINEA (BASDAI ≥ 4); C-OPTIMISE and COAST-Y (ASDAS ≥ 2.1 at 2 consecutive visits or ASDAS > 3.5 at any visit); Wetterslev et al. (BASDAI: change in BASDAI ≥ 20 and an absolute BASDAI ≥ 40 or clinical flare or MRI flare as pre-defined by the authors); GO-BACK (ASDAS ≥ 2.1 at 2 consecutive visits or an increase in ASDAS ≥ 1.1 at any visit); RE-EMBARK (ASDAS ≥ 2.1 at any visit)

Over the past 5 years, randomized strategy trials have occurred, including a comparison control arm. These trials have refined the criteria that allowed the discontinuation of biologics and mostly followed the ASDAS definitions of inactive disease (ASDAS < 1.3) or low disease activity (ASDAS < 2.1). The trials enrolled a larger sample size (range 53–153) compared to previous observational studies and included radiographic and non-radiographic axSpA patients. Most trials allowed for an instant discontinuation of the biologic without a tapering strategy. The flare rates were high and ranged between 53% and 85%. The definition of a flare was different between different trials (see Table 2). An international trial (COAST-Y) evaluated IL-17i (ixekizumab) instant discontinuation after 24 weeks of treatment [40]. Fifty-three axSpA patients in the withdrawal arm compared to 100 patients in the continuation arm were followed for 40 weeks. Forty-five percent (45%) of the patients had a reported flare as defined by ASDAS ≥ 2.1 at two consecutive visits or ASDAS > 3.5 at any follow-up visit. A recent observational study addressed the possibility of tapering the biologics before discontinuation as mentioned previously [27••]. In this study, axSpA patients who were considered to be in clinical remission (based on BASDAI < 40 and physician global score < 40) tapered TNFi over 48 weeks leading to biologic discontinuation. Only one patient (1%) could completely withdraw the treatment without flares, reflected by sustained BASDAI scores and lack of clinical or MRI flares as defined by the authors.

As more data emerge, axSpA disease flare with b-DMARD discontinuation is still substantially high. The studies that we discussed have several limitations that would require future well-designed protocols to address the feasibility of such an approach. The small sample size in most of these studies also makes it challenging to interpret the results with certainty. Most studies allowed for abrupt discontinuation of the b-DMARD after a relatively short period of treatment induction (less than 1 year in most studies). Convincing data on whether a b-DMARD taper can lead to successful discontinuation is currently lacking. Similarly, no data for successful b-DMARD withdrawal in patients considered to be in “prolonged remission” (more than 1 or 2 years) are available. Currently, no endorsed definition of “prolonged remission” exists. Further studies are also required to assess the possibility of withdrawal of IL-17i and JAKi in axSpA.

## Conclusion

Although the remission criteria for axSpA are not well defined, and there is considerable discordance between the physician’s and patient’s perception of remission, tapering TNFi by spacing or dose reduction may be a feasible option in certain patients with an acceptable disease state. The current data does not support the complete discontinuation of b-DMARDs owing to high flare rates. Future studies evaluating b-DMARDs (in addition to TNFi), comparing different regimens, with homogenous time in remission prior to taper, and longer disease duration are warranted to elucidate an effective drug tapering or withdrawal strategy.

## Data Availability

No datasets were generated or analysed during the current study.
